# Reemergence of Syphilis in Martinique, 2001–2008

**DOI:** 10.3201/eid1601.081730

**Published:** 2010-01

**Authors:** André Cabié, Bruno Rollin, Sandrine Pierre-François, Sylvie Abel, Nicole Desbois, Pascale Richard, Patrick Hochedez, Raphaëlle Théodose, Danielle Quist, Raymond Hélénon, Christian Derancourt, Annick Cavelier, Bernard Liautaud

**Affiliations:** Centre Hospitalier Universitaire de Fort-de-France, Fort-de-France, Martinique (A. Cabié, B. Rollin, S. Pierre-François, S. Abel, N. Desbois, P. Hochedez, R. Théodose, D. Quist, R. Hélénon, C. Derancourt, B. Liautaud); Clinical Research Center of French West Indies and French Guiana, Inserm CIE 802 (A. Cabié); Unité de Consultation et de Soins Ambulatoires Centre Médical Pénitentiaire de Fort-de-France (S. Abel, D. Quist); Etablissement Français du Sang, Fort-de-France (P. Richard); Vernes STD Clinic, Fort-de-France (R. Hélénon, A. Cavelier)

**Keywords:** Syphilis, Caribbean, outbreak, reemergence, Martinique, homosexuality, crack cocaine, bacteria, dispatch

## Abstract

Syphilis reemerged in Martinique in 2004 and initially affected 3 HIV-infected patients. By March 2008, syphilis was diagnosed for 37 men and 18 women. As of October 31, 2009, this outbreak had not yet been brought under control. It initially affected mainly men who had sex with men before it spread to heterosexual persons, minority group members, and crack cocaine users.

Syphilis was expected to reemerge in Martinique after outbreaks occurred in large western urban centers in 1998 (*1*,*2*), and cases were reported in Guadeloupe in 2001 (*3*). Soon after the first cases were diagnosed in Martinique, we conducted a study to determine whether these cases represented an outbreak and to identify demographic and social determinants (*4*) of this outbreak.

## The Study

In 2001, we increased syphilis screening at University Hospital in Fort-de-France, Martinique. Screening included use of the rapid plasma reagin (RPR) test and the *Treponema pallidum* hemaglutination assay (TPHA). All positive and discordant results were verified by using fluorescent treponemal antibody absorption, which detects *T*. *pallidum*–specific immunoglobulin (Ig) G and IgM. Darkfield microscopy was used whenever possible. We reviewed medical files of all patients who had received a diagnosis of syphilis during January 1, 2001–March, 31, 2008. Patients were included in the study if they had recent syphilis (primary, secondary, or early latent stage) as defined by the US Centers for Disease Control and Prevention (Atlanta, GA, USA) (*5*).

We investigated the yearly incidence of recent syphilis among HIV-infected patients treated at the infectious diseases unit of the hospital, at the Vernes Sexually Transmitted Disease (STD) Clinic (Fort-de-France, Martinique), and at anonymous voluntary counseling and testing clinics. We also obtained syphilis test results of all persons who were tested at the central laboratory of the hospital. Laboratory definition of active syphilis was an RPR titer >4 and a TPHA titer >80 for an initial screening test, or a 4-fold increase in RPR titer in samples after previously positive results. TPHA screening results for voluntary blood donors were collected at a blood bank.

Recent syphilis was diagnosed for 55 patients at University Hospital during 2001–2008 (Table 1). Patients (37 men and 18 women) had a median age of 41 years (interquartile range [IQR] 36–44 years). Twenty-one (57%) of 37 men were men who have sex with men (MSM), and 9 (43%) of 21 were bisexual. One fourth of the patients never used condoms. Of 36 patients questioned about oral sex, 30 admitted practicing oral sex, of whom only 2 (6.6%) always used condoms. Each patient’s median number of sexual partners during the previous 12 months was 2.5 (IQR 1.5–3.5).

**Table 1:** Characteristics of 55 patients with syphilis, University Hospital, Fort-de-France, Martinique, January 1, 2001– March 31, 2008*

Characteristic	Syphilis stage								Total
	Primary			Secondary			Early latent		
	HIV-positive	HIV-negative		HIV-positive	HIV-negative		HIV-positive	HIV-negative	
Total no. patients	1	11		24	9		4	6	55
Sex									
M	1	9		17	6		4	0	37
F	0	2		7	3		0	6	18
MSM	1	1		13	3		3	0	21
Crack cocaine use	0	6		8	6		0	3	23
Precarious conditions	0	5		6	5		0	1	17
HIV diagnosis									
Before syphilis	1	ND		18	ND		3	ND	22
Concomitant with syphilis	0	ND		6	ND		1	ND	7
Darkfield microscopy, no. positive/total	0/1	2/5		11/13	4/4		ND	ND	17/23
Modal RPR titer	16	8		128	16		32	32	32

*MSM, men who have sex with men; precarious conditions, >1 of the following: homelessness, lack of welfare, being followed-up in a psychiatry unit, mental deficiency, having paid sex, incarceration in correctional facility >2× in the past 5 years; ND, not determined; RPR, rapid plasma reagin.

One of the first patients to receive a diagnosis in 2004 reported >100 sexual partners, most during a recent stay in Paris. Primary, secondary, and early latent syphilis was diagnosed in 12, 33, and 10 patients, respectively, and 21 patients with secondary syphilis had genital lesions. Cholestatic hepatitis developed in 7 of 29 HIV-positive patients. Six patients had neurosyphilis or ophthalmic syphilis, all of whom also had secondary rashes.

Median RPR titer for the 55 patients was 32. Results of darkfield microscopy were positive for 17 (74%) of 23 patient specimens, 5 from genital mucosa and 12 from skin lesions. All patients had prevention counseling and were successfully treated with penicillin (except for 1 patient who was successfully treated with azithromycin). Seven relapses occurred. More than half of the patients were HIV positive (53%): 22 with a previous diagnosis of HIV infection and 7 with a new diagnosis of HIV infection at the time of syphilis diagnosis. Median duration of HIV infection was 48 months (IQR 21–91 months), and median CD4 lymphocyte count was 516 cells/μL (IQR 340–639 cells/μL).

At the time of syphilis diagnosis, 8 patients were receiving highly active antiretroviral therapy, and 4 had an HIV viral load <50 copies/mL. Twenty-three patients (42%) were crack cocaine users, and 17 patients (7 heterosexual men, 2 MSM, and 8 women) (31%) lived in precarious conditions (defined as >1 of the following: homelessness, lack of welfare, being followed-up in a psychiatry unit, mental deficiency, having paid sex, incarceration in a correctional facility >2× in the past 5 years).

**Figure 1:**
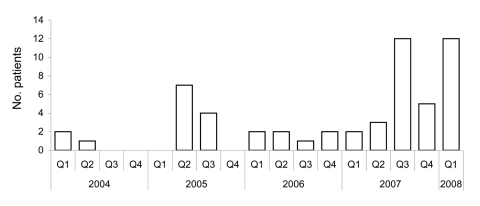
Patients with syphilis at University Hospital, Fort-de-France, Martinique, first quarter of 2004 through first quarter of 2008.

**Figure 2:**
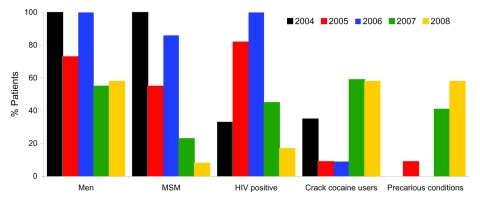
Frequency of syphilis in 55 patients, by group, Fort-de-France, Martinique, 2004–2008. MSM, men who have sex with men; precarious conditions, >1 of the following: homelessness, lack of welfare, being followed-up in a psychiatry unit, mental deficiency, having paid sex, incarceration in correctional facility >2× in the past 5 years.

The first cases of syphilis were diagnosed in 2004, with peaks in number of cases in 2005 and 2007–2008 (Figure 1). Demographic and social characteristics of patients changed rapidly. The first peak in 2005 included mostly HIV-infected MSM, and the second peak in 2007–2008 included almost as many women as men and a larger proportion of persons living in precarious conditions; crack-cocaine use and paid sex were more frequent, but HIV infection was lower (Figure 2).

During 2000–2007, the incidence of syphilis among HIV-infected patients increased from 0/1,000 to 12.1/1,000 patient-years (Table 2). A marked decrease in cases of recent syphilis diagnosed at the STD clinic occurred during 1987–1992, and <5 cases were recorded yearly during 2001–2007. Among persons tested for syphilis in anonymous voluntary counseling and testing clinics, 12 were diagnosed with recent syphilis during 2002–2007 (incidence <5 cases/year).

**Table 2:** Incidence of syphilis, Fort-de-France, Martinique, 2000–2007*

Characteristic	2000	2001	2002	2003	2004	2005	2006	2007
HIV-infected patients at University Hospital								
Total no. patients	479	492	508	544	564	659	724	746
MSM, no. (%)	107 (22)	108 (22)	110 (22)	126 (23)	124 (22)	160 (24)	184 (25)	185 (25)
Incidence of recent syphilis/ 1,000 patient-years, %	0	0	0	0	5.3	15.2	9.6	12.1
Incidence of recent syphilis/ 1,000 patient-years in MSM, %	0	0	0	0	24.2	31.2	21.7	27.1
Vernes STD Clinic								
Syphilis tests, no.	306	559	1,368	1,478	1,266	1,081	976	1,592
Recent syphilis, no.	0	1	2	3	0	0	3	4
A-VCT clinics								
Syphilis tests, no.	ND	ND	93	821	651	1,186	709	1,159
Recent syphilis, no.	ND	ND	0	1	2	4	2	3
University Hospital central laboratory								
Syphilis tests, no.	2,053	1,755	1,876	2,575	2,387	3,187	2,050	3,225
TPHA titer >80, no. (%)	189 (9.2)	171 (9.7)	182 (9.7)	235 (9.1)	200 (8.3)	258 (8.1)	114 (5.6)	217 (6.7)
RPR test titer >4/TPHA titer >80,† %	1.6	1.8	2.2	2.1	5.0	7.0	11.4	11.6
RPR test titer >8/TPHA titer >80, %	1.1	0.6	1.1	0.9	3.5	6.6	8.8	9.7
Blood donors								
Syphilis tests, no.	8,161	8,315	8,638	8,914	8,993	8,057	9,199	8,495
TPHA titer >80, no. (%)	45 (0.6)	49 (0.6)	39 (0.5)	42 (0.5)	30 (0.3)	31 (0.4)	33 (0.4)	27 (0.3)
RPR test titer ≥1, no. (%)	10 (0.1)	26 (0.3)	11 (0.1)	12 (0.1)	5 (0.06)	5 (0.06)	4 (0.04)	4 (0.05)
RPR test titer >4, no. (%)	1 (0.01)	1 (0.01)	1 (0.01)	1 (0.01)	0	0	1 (0.01)	0
RPR test titer >4/TPHA titer >80,† %	2.2	2.0	2.6	2.4	0	0	3.0	0

*MSM, men who have sex with men; STD, sexually transmitted disease; A-VCT, anonymous voluntary counseling and testing; TPHA, *Treponema pallidum* hemaglutination assay; RPR, rapid plasma reagin; †Defines active syphilis.

Among blood donors, incidence of positive TPHA results was 0.3%–0.6% during 2000–2007, and an incidence of 1.04% was observed in 1999. However, the outbreak was too limited to have affected this group. The incidence of active syphilis as a proportion of all cases reported to the central laboratory database increased from 1.6% to 11.6% during 2000–2007 (Table 2).

## Conclusions

Infectious syphilis had become so rare in France and in the French Caribbean islands in the 1990s that mandatory notification of this disease was canceled in 2000. That same year, outbreaks were reported by several STD clinics in France (*6*). These outbreaks followed reports of outbreaks among MSM in major urban centers in the United States (*2*,*7*), Canada, Europe, Australia, and New Zealand (*4*). Three epidemiologic profiles of syphilis patients were recently defined on the basis of social determinants (*4*): general populations in developing countries, minority populations with a low socioeconomic status in industrialized countries, and MSM.

In 2001, an outbreak of 58 cases of recent syphilis diagnosed during 1993–2001 (38 cases in 2001) was reported in Guadeloupe (*3*). This outbreak occurred mainly (89%) in the population living in precarious social and economic conditions; 21% had a history of recent imprisonment, and 50% used crack cocaine and had paid sex. The M:F ratio increased from 0.81:1 to 1.37:1. Twenty-six percent of the patients were HIV positive.

A limited but uncontrolled syphilis outbreak is ongoing in Martinique. It started with an MSM epidemiologic profile, then shifted to persons within a specific heterosexual group that included crack cocaine users. This shift is similar to that reported for HIV infection in the Caribbean in the 1980s (*8*–*11*). Bisexuality may play a role by linking different populations during HIV and syphilis epidemics (*8*,*11*,*12*).

Although our study was relatively small and retrospective, it offered a unique opportunity to observe an emerging outbreak in a relatively isolated population of 400,000 in a small geographic area. Early detection also provides a unique opportunity for therapeutic and preventive intervention.

Interpretation of syphilis test results is often difficult in asymptomatic patients, especially when new-generation tests are used in low-prevalence countries (*13*). In the Caribbean, although yaws was largely eradicated in the 1960s, small outbreaks continue to be reported, as in Martinique from 1974 through 1985. This finding further complicates interpretation of screening results, particularly for older patients.

Syphilis outbreaks will be difficult to detect in regions of the Caribbean where the disease is highly endemic and surveillance is poor. Outbreaks of syphilis and congenital syphilis were reported in Trinidad and Tobago and Jamaica in the 1990s (*14*,*15*). Control of outbreaks requires coordinated public health interventions, including new preventive strategies specific for high-risk groups. Preventive messages must be culturally appropriate and must underline the risk for STD transmission by oral sex.
